# Characterization of a Novel Interaction between Bcl-2 Members Diva and Harakiri

**DOI:** 10.1371/journal.pone.0015575

**Published:** 2010-12-30

**Authors:** Lorenzo Sborgi, Susana Barrera-Vilarmau, Patricia Obregón, Eva de Alba

**Affiliations:** Centro de Investigaciones Biológicas, Consejo Superior de Investigaciones Científicas, Madrid, Spain; National Institute for Medical Research, United Kingdom

## Abstract

Interactions within proteins of the Bcl-2 family are key in the regulation of apoptosis. The death-inducing members control apoptotic mechanisms partly by antagonizing the prosurvival proteins through heterodimer formation. Structural and biophysical studies on these complexes are providing important clues to understand their function. To help improve our knowledge on protein-protein interactions within the Bcl-2 family we have studied the binding between two of its members: mouse Diva and human Harakiri. Diva has been shown to perform both prosurvival and killing activity. In contrast, Harakiri induces cell death by interacting with antiapoptotic Bcl-2 members. Here we show using ELISA and NMR that Diva and Harakiri can interact in vitro. Combining the NMR data with the previously reported three-dimensional structure of Diva we find that Harakiri binds to a specific region in Diva. This interacting surface is equivalent to the known binding area of prosurvival Bcl-2 members from the reported structures of the complexes, suggesting that Diva could function at the structural level similarly to the antiapoptotic proteins of the Bcl-2 family. We illustrate this result by building a structural model of the heterodimer using molecular docking and the NMR data as restraints. Moreover, combining circular dichroism and NMR we also show that Harakiri is largely unstructured with residual (13%) α-helical conformation. This result agrees with intrinsic disorder previously observed in other Bcl-2 members. In addition, Harakiri constructs of different length were studied to identify the region critical for the interaction. Differential affinity for Diva of these constructs suggests that the amino acid sequence flanking the interacting region could play an important role in binding.

## Introduction

Programmed cell suicide known as apoptosis controls cell homeostasis and is thus central to the life cycle of multi-cellular organisms [1]. Proteins of the Bcl-2 family are key regulators of apoptotic mechanisms by mediating in an intricate network of interactions between pro- and antiapoptotic members that eventually lead to the activation of caspases, the true apoptosis executors [2]–[3]. Bcl-2 proteins share low sequence homology in small stretches of amino acids named Bcl-2 homology (BH) domains. Members that promote cell survival (e.g. Bcl-2, Bcl-X_L_, Bcl-w, Mcl-1, BFL-1) contain four BH domains (BH1-BH4), whereas members with killing activity can share homology either in three BH domains or solely in the BH3 region (the BH3-only subfamily). As a response to death stimuli, BH3-only proteins form heterodimers with prosurvival members, thus antagonizing their function [4]–[7]. Reported evidence indicates that peptides of ∼16–25 amino acids comprising the BH3 domain of BH3-only proteins suffice for heterodimer formation [8]. Therefore, most of the structural information currently known on BH3-only proteins is centered at BH3 peptides. All known three-dimensional (3D) structures of complexes between prosurvival Bcl-2 members and these peptides show that the latter adopt α-helical structure and are located in a hydrophobic groove of the prosurvival protein surface [8]–[9]. However, BH3 peptides have been shown to behave like random coils in isolation [9], and experimental evidence together with prediction programs support that several BH3-only proteins are intrinsically disordered [10]. Thus, it has been suggested that additional energetic factors besides specific intermolecular interactions likely play a role in this peculiar binding process [9].

The dysfunction of apoptotic mechanisms has been pointed as a hallmark of cancer. In particular, tumor cells overexpress prosurvival Bcl-2 members and tumor suppressor p53 fails at activating several BH3-only proteins conferring death resistance to cancer cells [11]. These findings have both increased interest in the use of BH3-only proteins as scaffolds for drug design [12]–[14] and targeted research at the detailed understanding of Bcl-2 interactions. Recent work in this direction has shown that antiapoptotic Bcl-2 members can bind preferentially particular subsets of BH3-only proteins [15]–[17]. This selectivity has been related to differential apoptotic response [16], [17]. However, the conclusions derived from these studies are at variance likely because of the complexity of the molecular mechanisms involved as well as the need to compare in vitro and in vivo data. Additional work is thus necessary to fully understand Bcl-2 interactions and their relation to programmed cell death. To gain insight into the structural and biophysical factors involved in Bcl-2 protein-protein binding, we report here the characterization of a novel interaction between the BH3-only protein Harakiri and the Bcl-2 member Diva (also called Boo).

Harakiri localizes in membranes and exerts proapoptotic activity by interacting with survival Bcl-X_L_ and Bcl-2 [18]. Harakiri has not been characterized at the structural level except for its C-terminal sequence (∼30 amino acids), which is known from low-resolution techniques to adopt α-helical conformation in model membranes [19]. Diva has also been found predominantly in membranes [20]–[21]. However, little functional data on Diva is available. Specifically, previous independent reports indicate that Diva can have both pro- or antiapoptotic function [20]–[21]. Diva has also been reported to bind antiapoptotic Bcl-X_L_, and the proapoptotic Bcl-2 members Bik and Bak, according to co-immunoprecipitation assays [21]. In contrast, binding studies using isothermal titration calorimetry indicate that Diva does not bind peptides comprising the BH3 region of several proapoptotic Bcl-2 proteins, including Bak and Harakiri [22]. On this basis it has been suggested that Diva is not functionally equivalent to other Bcl-2 proteins [22]. However, the 3D structure of Diva is very similar to the known structures of other Bcl-2 members [22].

Here we show using ELISA and NMR that Diva and Harakiri can interact in vitro. Our NMR data combined with the recently reported structure of Diva [22] indicate that the interaction involves in Diva's surface the same groove previously observed in all other known structures of antiapoptotic/BH3-peptide complexes, indicating that binding is specific. To illustrate the formation of the complex a 3D structural model of the heterodimer is built using molecular docking and the NMR data as restraints. Altogether, these results suggest that at the structural level Diva binds death-inducing Harakiri in a fashion similar to other antiapoptotic Bcl-2 proteins. In addition, structural studies on Harakiri were carried out using NMR and circular dichroism. The data show that Harakiri is largely unstructured with only a small population of residual α-helical conformation. This result indicates that Harakiri is an intrinsically disordered protein like several other members of the BH3-only subfamily [10]. As BH3-derived peptides in isolation show little structure [9] whereas they form a helix when bound to the prosurvival protein, it is plausible that structure formation in the peptide is connected to binding [9], [10]. Thus, using NMR titration experiments we estimated an apparent dissociation constant of the complex assuming a simple model that takes into account Harakiri folding upon binding. In addition, by studying the binding to Diva of Harakiri constructs of different length we identify the critical region for binding in Harakiri and observe that affinity increases for constructs longer than this region, suggesting that the flanking sequence can influence binding.

## Results

### Interaction between Diva and Harakiri detected by ELISA

**Figure 1:**
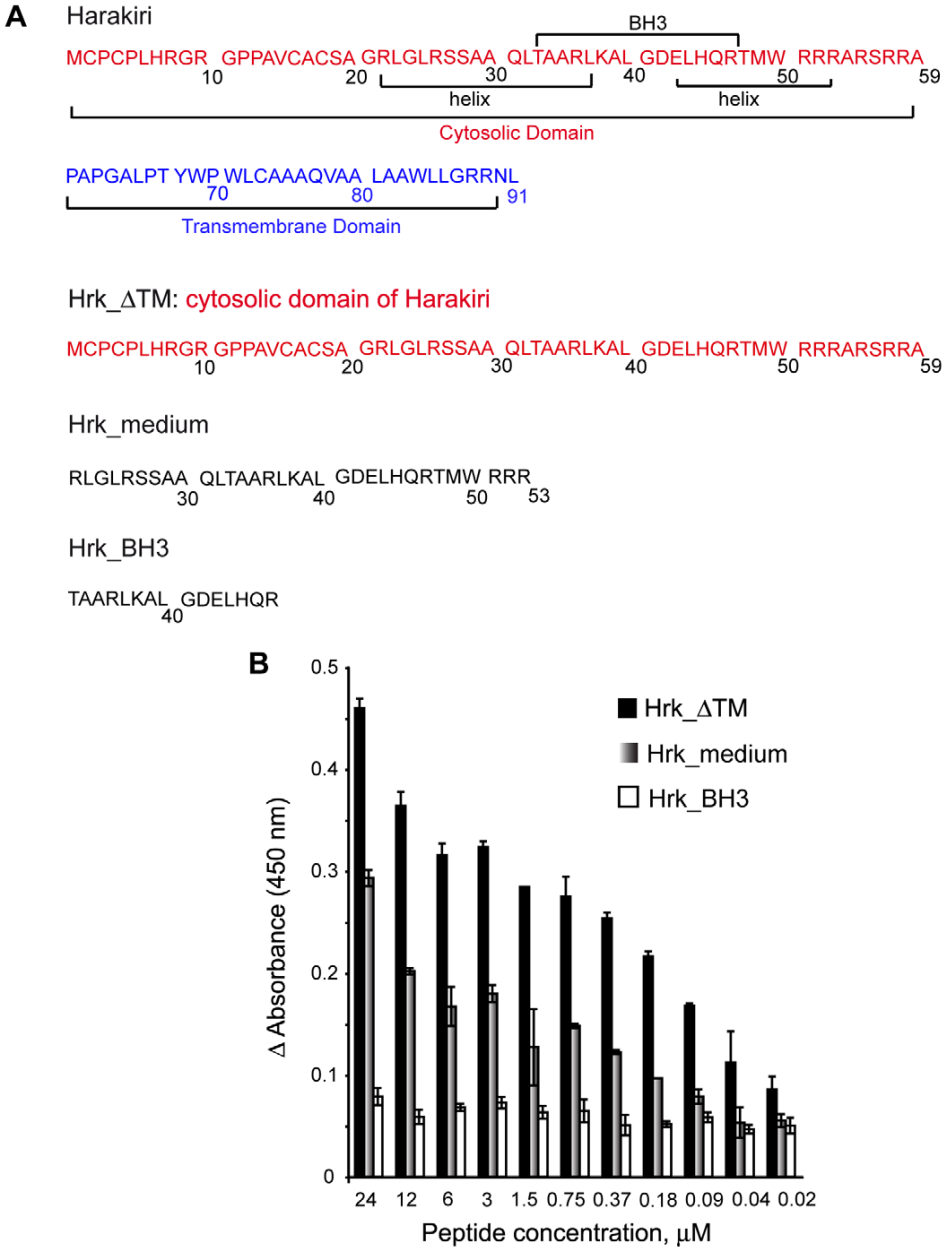
Harakiri constructs and ELISA binding. (A) One-letter code amino acid sequence of human Harakiri and constructs studied. The BH3 region, the transmembrane domain and the predicted α-helical segments are indicated. (B) Difference in ELISA absorbance relative to the control for the binding of Diva to Hrk_ΔTM, Hrk_medium and Hrk_BH3 vs. fragment concentration. Shown values are average of two measurements. Thin bars represent the standard deviation. The absorbance value for the control is 0.12±0.01.

The interaction between Harakiri and Diva was studied using three Harakiri constructs of different length all including the BH3 region (**Fig. 1A**): the entire cytosolic domain of Harakiri encompassing residues 1 to 59 (Hrk_ΔTM) [18], a 32 residue-long peptide comprising the BH3 domain flanked by two segments predicted to have α-helical propensity according to the program PredictProtein [23] (Hrk_medium), and a 16 amino acid-long peptide spanning the BH3 domain alone (Hrk_BH3).

ELISA was used to test the binding capabilities of the three constructs to Diva. Hrk_ΔTM and Hrk_medium show significant levels of interaction, with the entire cytosolic domain displaying higher binding affinity (**Fig. 1B**). Interaction levels typically decrease with peptide concentration as expected (**Fig. 1B**). In contrast, protein-binding levels are negligible for the shortest fragment Hrk_BH3 and do not show any dependence with peptide concentration, suggesting that the observed residual signal corresponds to background levels (**Fig. 1B**). Altogether, ELISA data indicate that the length of the Harakiri constructs has a significant effect on binding to Diva.

### NMR characterization of Diva/Harakiri interaction

**Figure 2:**
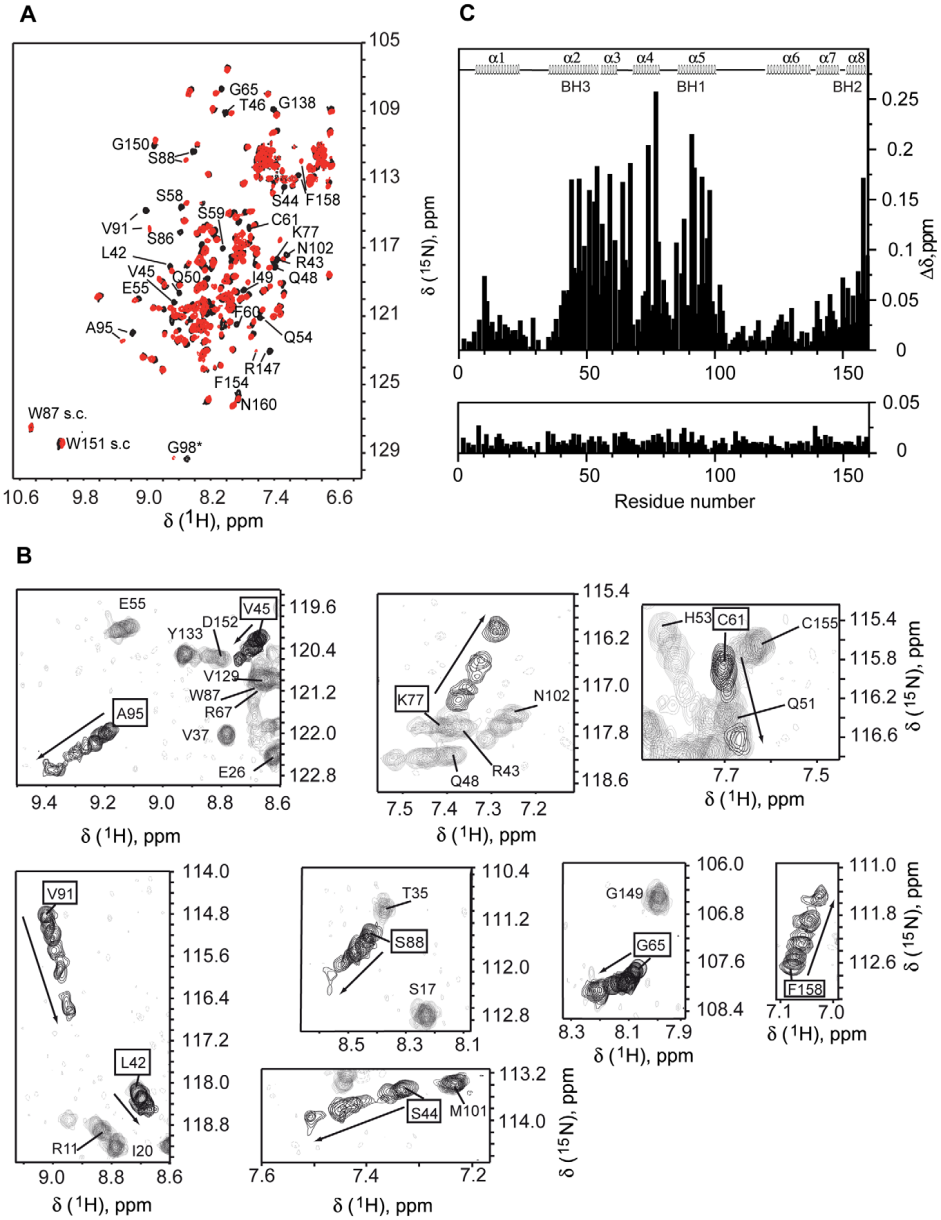
Interaction between Diva and Harakiri by NMR. (A) [^1^H-^15^N]-HSQC spectra of ^15^N,^13^C-labeled Diva (black), and Diva (0.12 mM) in the presence of unlabeled Hrk_ΔTM (0.85 mM) (red). Several residues undergoing large perturbations are indicated. The asterisk indicates a folded peak. Trp side chains are labeled as “s.c.”. (B) Superposition of regions of Diva [^1^H-^15^N]-HSQC spectra resulting from the titration with Hrk_ΔTM. Residues used in the estimation of the K*_d_app* are boxed and the linewidth of the corresponding signals has been increased relative to the others for clarity. Shown spectra correspond to 9 titration points out of the 16 measured at the following values of Hrk_ΔTM concentration (mM): 0, 0.08, 0.16, 0.29, 0.44, 0.73, 0.85, 1.60, 1.80. Some panels look particularly crowded because of the superposition of a total of 9 spectra. The noise level of the spectrum corresponding to the titration at 1.80 mM Hrk_ΔTM was reduced for clarity as the threshold level had to be lowered to properly determine the chemical shift of the significantly broaden signals. (C) Upper panel: chemical shift differences (Δδ = {[Δδ^1^H)]^2^+[Δδ(^15^N)/5]^2^}^1/2^) between spectra of Diva and a mixture of Diva (0.12 mM) and Hrk_ΔTM (0.85 mM) versus Diva's residue number. Lower panel: chemical shift changes between spectra resulting from Diva/Hrk_ΔTM (0.18 mM/0.37 mM) and Diva/Hrk_medium (0.1 mM/1 mM) mixtures vs. Diva's residue number. The position of the α-helices and the BH domains is indicated.

**Figure 3:**
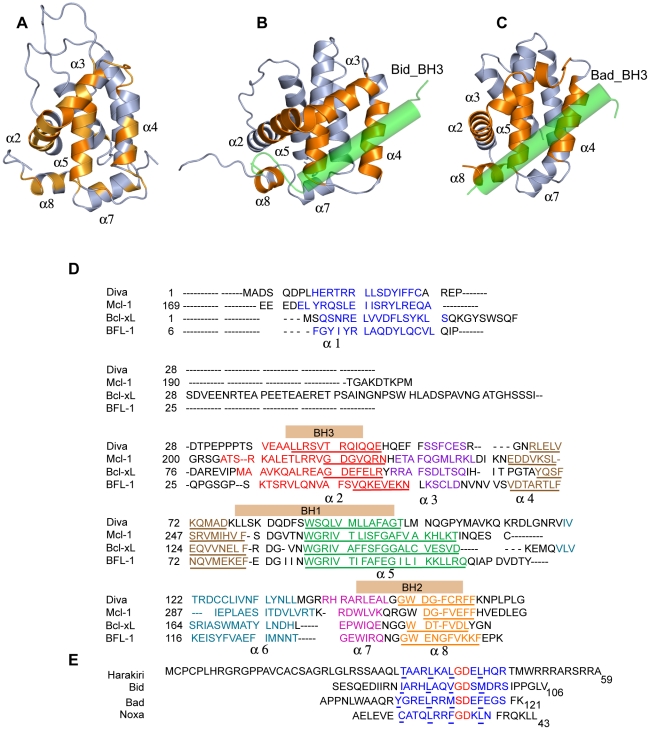
Interacting region mapped in the 3D structure of Diva. Comparison to other prosurvival/BH3_peptide complexes. (A) Ribbon diagram of the 3D structure of Diva (pdb ID 2KUA) showing in orange the position of residues with chemical shift perturbations upon binding to Hrk_ΔTM. Light and dark orange indicate perturbations between 0.04 and 0.1 ppm, and larger than 0.1 ppm, respectively. Helices involved in the interaction are numbered. (B,C) Ribbon diagram of human Mcl-1/Bid_BH3 (pdb ID 2KBW) (B) and mouse Bcl-X_L_/Bad_BH3 (pdb ID 2BZW) (C) complexes. The peptides are shown as translucent green cylinders. The orientation of Diva, Mcl-1 and Bcl-X_L_ is equivalent. Helices forming the typical binding pocket are colored in dark orange and numbered. Fig. 3A,B,C were created with PyMOL [43]. (D) Secondary structure and sequence alignment of mouse Diva (Q9Z0F3), human Mcl-1 (Q07820), mouse Bcl-X_L_ (Q64373) and human BFL-1 (Q16548) used in the structural studies of the complexes with Harakiri, Bid (pdb ID 2KBW), Bad (pdb ID 2BZW) and Noxa (pdb ID 3MQP) respectively. Helices are color coded and numbered from 1 to 8. BH domains appear as bars. The amino acid sequences correspond to those of the structural studies, and thus do not show the C-terminal TM helix. Residues involved in the interacting surface with the cytosolic domain of Harakiri and the other BH3 peptides are underlined. (E) Sequence alignment of the cytosolic domain of Harakiri and the BH3 peptides used in the structural studies mentioned in D. The BH3 domains are colored in blue. The conserved small residue (Ser/Gly) and Asp residue in the BH3 domain are shown in red. The four conserved hydrophobic residues in the BH3 domain of Harakiri and those known to form part of the contact area in the complexes are underlined.

We further studied the interaction between Diva and Harakiri by NMR, as this is the technique of choice to identify at the atomic level structural changes associated to protein interactions [24]. Amide [^1^H,^15^N]-HSQC experiments [25] of ^15^N-labeled Diva were recorded upon the addition of unlabeled Hrk_ΔTM. Significant changes in NMR signals were observed, thus confirming the interaction (**Fig. 2A**). Chemical shift assignments of the backbone amide ^1^H and ^15^N resonances were obtained for each titration point (**Fig. 2B**). Residues with the largest chemical shift perturbations (>0.1 ppm) are mainly located in the regions 44–67, 74–78, 85–98 and 155–160 (**Fig. 2C**) indicating their participation in the binding interface. According to the secondary structure of Diva [26] these residues belong to helices 2, 3, 4, 5 and 8 (**Fig. 2C**). In addition, the mapping of the chemical shift perturbation data on the 3D structure of Diva [22] reveals that the binding region is equivalent to that observed in all known 3D structures of complexes between antiapoptotic Bcl-2 members and BH3-derived peptides [8], [9], [27], [28]. This result is illustrated in **Figure 3** using as examples the 3D structures of the complexes human Mcl-1/Bid_BH3 peptide [28] and mouse Bcl-X_L_/Bad_BH3 peptide (pdb ID 2BZW) (**Fig. 3B,C**). The 3D structure of human Mcl-1/Bid_BH3 peptide was selected because Mcl-1 is the closest structural analogue to Diva according to the Dali server [29] and the peptide is the longest reported up to date for these complexes (35 residues), thus more similar to the Diva/Hrk_ΔTM interaction. In addition, the structure of mouse Bcl-X_L_/Bad_BH3 peptide complex was included to help illustrate that heterodimers of human and mouse species are equivalent at the structural level (**Fig. 3**). The interaction between prosurvival Bcl-2 members and BH3-only proteins is specific in that binding always involves the BH3 domain of the BH3-only protein and helices 2, 3, 4, 5 and 8 of the prosurvival partner. According to the chemical shift perturbation data the Diva/Hrk_ΔTM interaction shares the same type of specificity as illustrated in the sequence alignments shown in **Figure 3D&E**.

**Figure 4:**
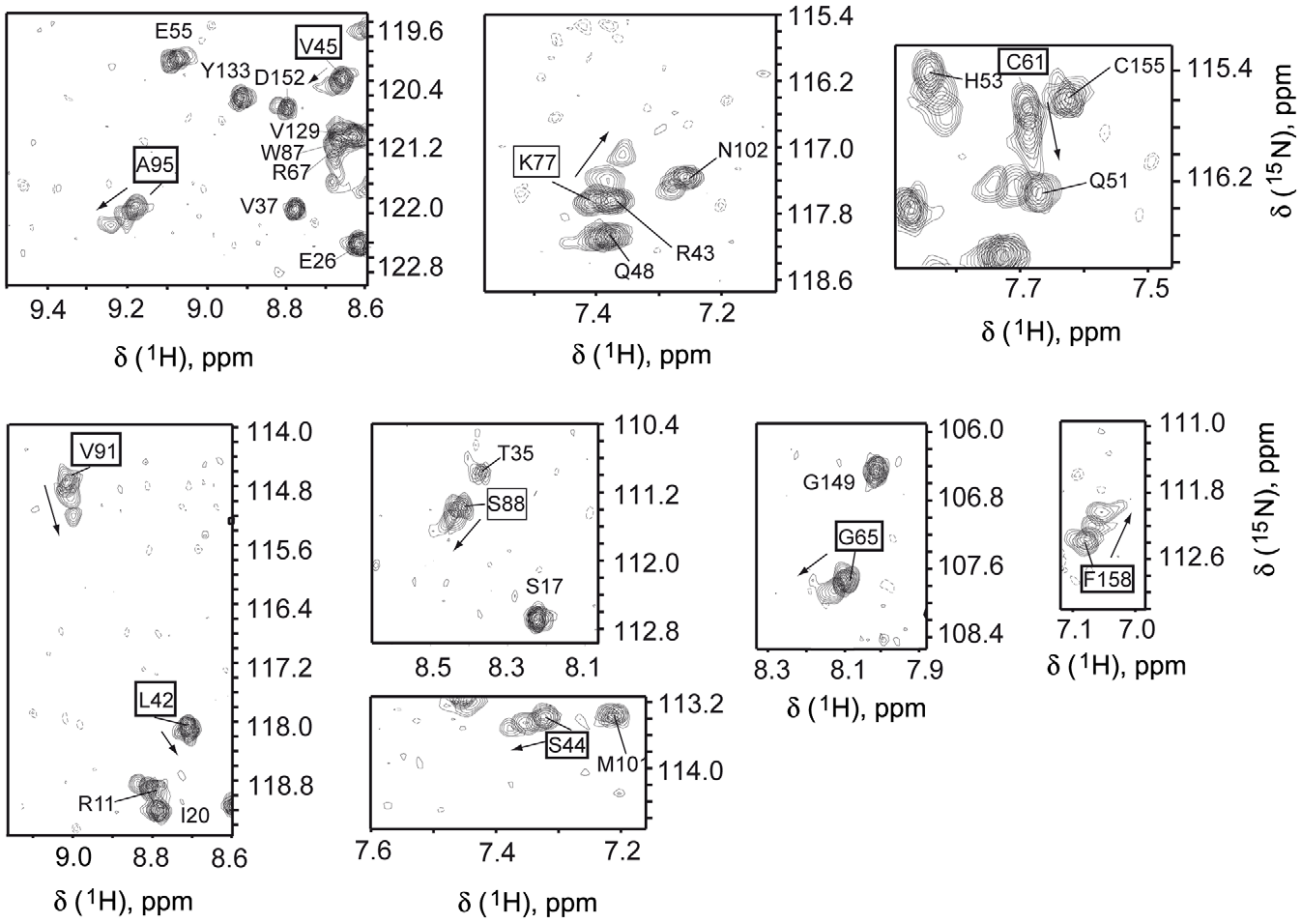
Interaction between Diva and Hrk_medium by NMR. Superposition of regions of Diva [^1^H-^15^N]-HSQC spectra resulting from the titration with Hrk_medium. Signals of residues boxed in Fig. 2B are shown here for comparison. Spectra correspond to the following Hrk_medium concentration values (mM): 0, 0.12, 0.5, 1.0.

The interaction of Diva with the shorter fragments Hrk_medium and Hrk_BH3 was also studied by NMR. For the shortest fragment Hrk_BH3, no chemical shift changes in the [^1^H,^15^N]-HSQC spectrum of Diva were observed even at a Diva/Hrk_BH3 ∼1∶15 molar ratio (Diva at 0.1 mM and Hrk_BH3 at 1.5 mM) (data not shown), suggesting the absence of the interaction. In contrast, Diva's NMR spectrum varies upon the addition of Hrk_medium (**Fig. 4**). The spectrum of a mixture of Diva and Hrk_medium at ∼1∶10 molar ratio (Diva 0.1 mM and Hrk_medium 1 mM) shows changes almost identical to those observed for the binding to Hrk_ΔTM at ∼1∶2 molar ratio (Diva 0.18 mM, Hrk_ΔTM 0.37 mM) (**Fig. 2C**, lower panel). Therefore, there is very good agreement between the NMR and ELISA results on weaker binding observed for Hrk_medium relative to the entire cytosolic domain, and negligible binding for Hrk_BH3 (**Fig. 1B**). Moreover, the identical NMR spectra obtained for Diva/Hrk_ΔTM and Diva/Hrk_medium mixtures (**Fig. 2C**, lower panel) indicate that the binding interface of both complexes is analogous.

### Hrk_ΔTM is largely disordered and shows residual α-helical structure

**Figure 5:**
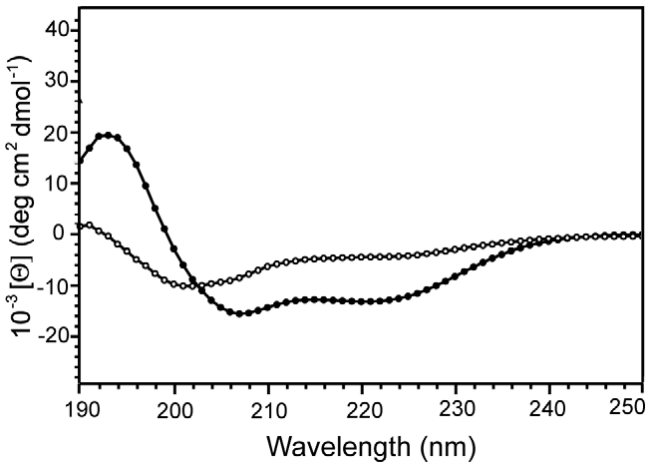
Circular dichroism spectra of Hrk_ΔTM. Far–UV CD spectra of Hrk_ΔTM in the absence (white circles) and presence (black circles) of 35% (v/v) trifluoroethanol. The percentage of α-helical population calculated as described in Materials and Methods is 13% and 35%, respectively. The minimum of the curve at 222 nm is characteristic of helix formation.

**Figure 6:**
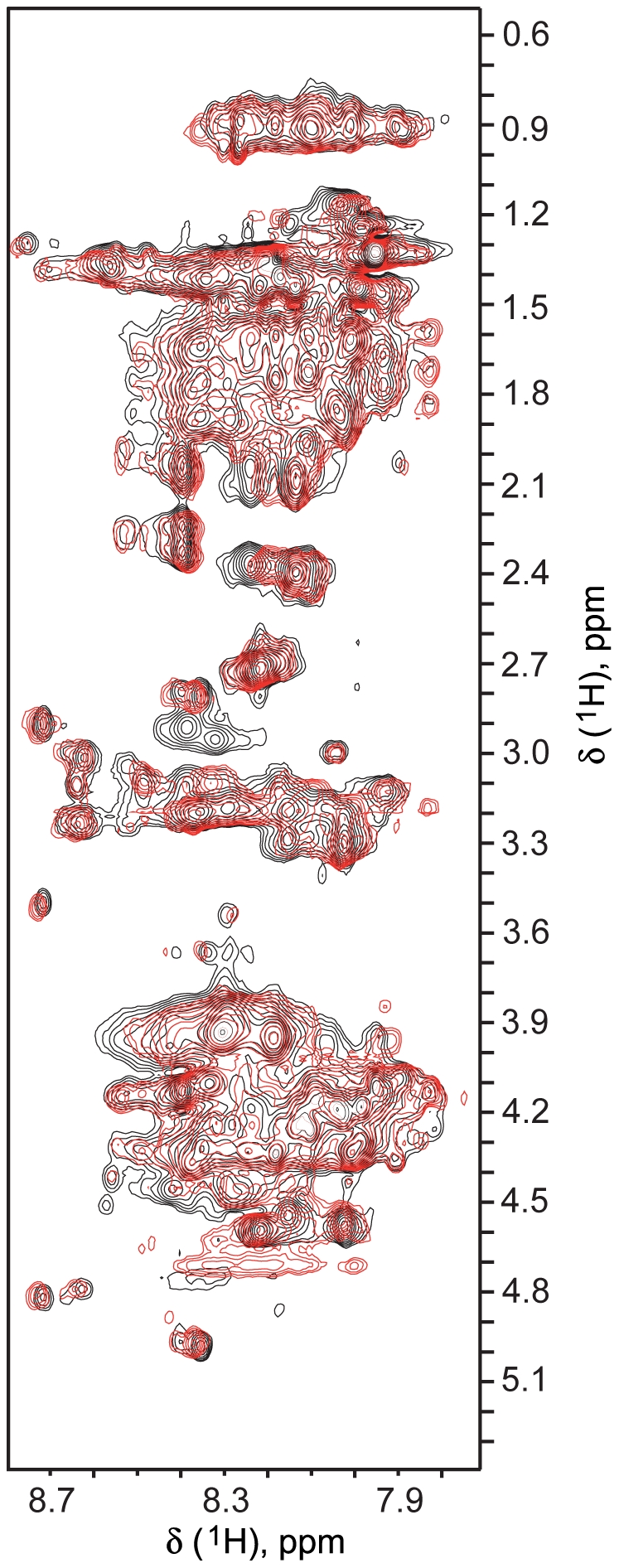
Changes in the NMR spectra of Hrk_ΔTM resulting from the interaction with Diva. ^1^H_amide_-^1^H_aliphatic_ region of TOCSY spectra of Hrk_ΔTM (black) and Hrk_ΔTM/Diva mixture (0.85 mM/0.12 mM) (red). The small dispersion in ^1^H amide chemical shifts, the absence of signals for methyl groups close to 0 ppm and the severe signal overlap are characteristic of disordered proteins. Changes in the chemical shifts of Hrk_ΔTM upon the addition of Diva likely result from an overall structural rearrangement toward the helical conformation in addition to the interaction with Diva. Signals in the [^1^H-^1^H]-TOCSY spectrum of the mixture are observed only for Hrk_ΔTM because of the low concentration of Diva relative to Hrk_ΔTM and Diva's broader signals, most likely at the noise level according to its larger molecular weight (∼18 kDa for Diva, ∼6 kDa for Hrk_ΔTM).

Circular dichroism (CD) experiments were performed on Harakiri's cytosolic domain to obtain information on its conformational behavior. The CD data indicate that Hrk_ΔTM adopts α-helical structure with ∼13% population in aqueous milieu that increases to ∼35% in the presence of the secondary structure enhancer trifluoroethanol (**Fig. 5**). Thus, the cytosolic domain of Harakiri is largely unstructured. Consequently, the NMR spectra of Hrk_ΔTM show small amide ^1^H chemical shift dispersion (from ∼7.9 to 8.7 ppm) and severe signal overlap (**Fig. 6**). These spectral features together with the absence of methyl signals at ∼0 ppm are characteristic of disordered proteins, in agreement with the CD data. Similar conformational behavior has been observed for BH3-derived peptides [9] and other members of the BH3-only subfamily [10]. For instance, the BH3-only proteins Bim, Bad, and Bmf have been found to be unstructured. In particular, Bim undergoes partial folding upon binding to Bcl-2 members [10]. The relation between function and disorder is a distinctive feature of natively unfolded proteins [30], thus several BH3-only members have been suggested to be intrinsically disordered [10]. Moreover, coupled folding and binding, a mechanism shown to be followed by some natively unfolded proteins [31], has been proposed for BH3-only proteins that are disordered in the unbound state and adopt helical structure when complexed [10]. The residual α-helical structure and sensitivity to trifluoroethanol of Hrk_ΔTM indicate its propensity to fold as a helix, thus suggesting a connection between folding and binding to Diva. The observed changes in the signals of the NMR spectrum of Hrk_ΔTM upon the interaction (**Fig. 6**) are small because chemical shifts are averaged by the populations of the bound and unbound forms, and the latter predominates under the conditions used (0.85 mM Hrk_ΔTM and 0. 12 mM Diva). The upper limits for the bound and unbound forms are 0.12 mM and 0.73 mM, respectively. The observed chemical shift changes could result from both the formation of the helix and the interaction itself. However, further analysis of the spectra is significantly complicated by the small chemical shift dispersion and signal overlap (**Fig. 6**).

### Structural model of Diva/Harakiri heterodimer

**Figure 7:**
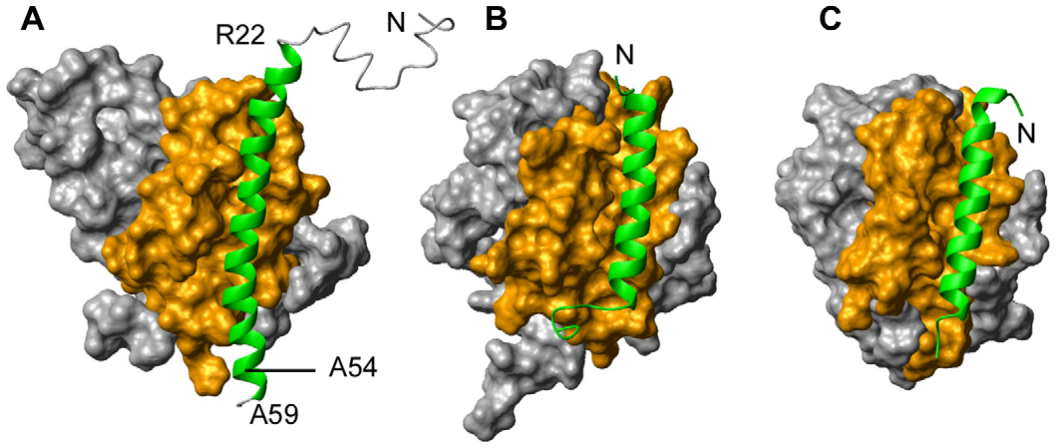
3D model of the Diva/Harakiri heterodimer compared to other prosurvival/BH3_peptide complexes. (A,B,C) Surface and ribbon representation of the Diva/Hrk_ΔTM heterodimer (A), Mcl-1/Bid_BH3 (pdb ID 2KBW) (B) and Bcl-X_L_/Bad_BH3 (pdb ID 2BZW) (C) complexes. The interacting surface in Diva and the other prosurvival proteins is colored in orange. The BH3-only protein fragments are shown as green ribbons and the N-termini are indicated. Residues Arg 22, Ala 54 and Ala 59 in Hrk_ΔTM are indicated to show that the N and C-terminal regions of the cytosolic domain of Harakiri are outside the critical binding region. The orientation of the three complexes is equivalent. Fig. 7 was created with MOLMOL [42].

To illustrate the interaction between Diva and Harakiri we built a model of the complex using molecular docking and the chemical shift perturbation data (**Fig. 2C**). In the 3D structures of all known heterodimers the BH3-peptide forms a helix [8], [9], [28]. Therefore, based on the residual α-helical structure of the entire cytosolic domain of Harakiri, a 3D model of the helix was obtained using a protein structure prediction program and the structure of the helix formed by a BH3-peptide bound to a Bcl-2 member as template (Materials and Methods). It is noteworthy that the modeled helix spans residues 23 to 59, which includes the BH3 region and the predicted helices (**Fig. 1A**), leaving the N-terminal 22 residues with no regular secondary structure. This 3D model together with the reported structure of Diva [22] were used in the molecular docking protocol, which also included information from the NMR data on the interacting surface in Diva as experimental restraints (Materials and Methods). The complexity of Harakiri NMR spectra precludes the identification of residues directly involved in the interaction, and thus residues 22–53 (Hrk_medium), which are known to be critical for binding from the NMR and ELISA data, were all input as participating residues in the docking protocol. The resulting 3D model of the heterodimer shows a binding cleft formed by helices 2–5 and 8 that is occupied by the modeled α-helical cytosolic domain of Harakiri (**Fig. 7A**). This cleft is topologically similar to all other known heterodimers of the family (examples are shown in **Fig. 7B,C**), which suggests that Diva is capable of interacting at the structural level like other antiapoptotic Bcl-2 members.

The structural model of the Diva/Harakiri heterodimer also explains the almost identical chemical shift perturbation data obtained for the binding of Diva to Hrk_ΔTM and Hrk_medium (**Fig. 2C**, lower panel). Residues 1–21 and 54–59 of Hrk_ΔTM flanking the sequence of Hrk_medium (**Fig. 1A**) fall outside the binding area as shown in **Fig. 7A**. As Hrk_ΔTM binds with higher affinity than Hrk_medium, the structural model suggests that the flanking sequences, albeit located outside the relevant interacting region in Harakiri, could play a role in binding.

Mouse Diva and human Harakiri are better studied than their human and mouse homologues, respectively. Thus, for practical reasons here we have characterized the hybrid interaction between the two proteins. The question that follows is whether binding between the intraspecies proteins is similar. There are several facts suggesting that the interaction will conserve the same overall structural features. For instance, the 3D structures in **Fig. 7B&C**, which correspond to human and mouse heterodimers are equivalent at the structural level. Moreover, the hybrid model built for Diva/Hrk_ΔTM is structurally compatible with them (**Fig. 7**). In addition, the amino acid sequences of Hrk_ΔTM and Hrk_medium in human and mouse are 75% identical, also suggesting a similar binding behavior. Furthermore, the four conserved hydrophobic residues in the BH3 domain known to anchor the helix to the antiapoptotic protein groove [**8**] (residues T33, L37, L40 and L44 for Harakiri) are identical both in the human and mouse species. Together, these considerations and the observed specificity of the interaction suggest that intraspecies protein binding between Diva and Harakiri is analogous at the structural level.

### Apparent binding affinity between Diva and Harakiri

NMR titration experiments on Diva upon binding to the cytosolic domain of Harakiri were performed to estimate an apparent dissociation constant of the complex (**Fig. 2B**). However, we have shown that Harakiri is largely unstructured before binding and as already established for BH3-peptides [8], [9], we propose that it adopts helical structure upon complex formation. It has been previously reported that coupled folding-and-binding is not realistically represented by the conventional two-state process [31]. Therefore, in an attempt to obtain an approximate value of the dissociation constant of the Diva/Hrk_ΔTM complex we have assumed the simplest binding model that takes into account Hrk_ΔTM folding/unfolding equilibrium:
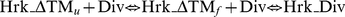



Where Hrk_ΔTM*_u_* and Hrk_ΔTM*_f_* are the unfolded and folded forms of Hrk_ΔTM, respectively. Div is the unbound form of Diva and Hrk_Div is the complex (see Materials and Methods).

**Figure 8:**
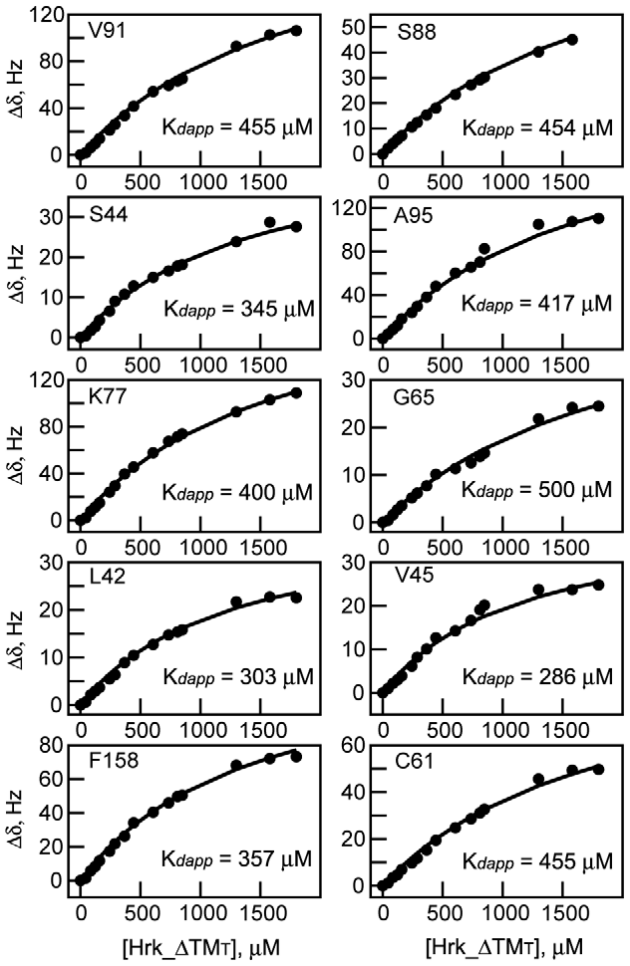
Determination of the apparent dissociation constant of the Diva/Harakiri complex. Chemical shift changes of signals in the [^1^H-^15^N]-HSQC spectra of Diva shown in Fig. 2B vs. the total concentration of Hrk_ΔTM (black circles). Fittings of the experimental data to equation (11) (Materials and Methods) are shown with a continuous line. The corresponding residues and individually measured K*_d_app* values are indicated. The concentration of Hrk_ΔTM (mM) for each point is: 0, 0.04, 0.08, 0.12, 0.16, 0.24, 0.29, 0.37, 0.44, 0.61, 0.73, 0.81, 0.85, 1.30, 1.60, 1.80. Diva's NMR signals undergo significant broadening for the last three titration points at the largest Hrk_ΔTM concentration, and thus the error in chemical shift determination for these points is likely larger.

The NMR titration experiments (**Fig. 2B**) depend on both the dissociation constant of the complex (K*_d_app*) and the unfolding/folding constant (K*_u_*). These two processes cannot be separated in the overall chemical shift change resulting from complex formation. Therefore, for proper fitting of the data the K*_u_* value was fixed based on the structure population obtained from the CD experiments. The values of *K_d_app* and K*_u_* strongly depend on each other thus, upper and lower limits of *K_d_app* can be obtained considering two possible scenarios in the folding/unfolding equilibrium of Hrk_ΔTM. On the one hand we assumed that the observed 13% of helical population in the CD spectrum of the 59-residue long construct is localized in the critical binding region from residues 22–53 (Hrk_meduim), which leads to a K*_u_* value of ∼3.1. By fixing this parameter, changes in the chemicals shifts of Diva (**Fig. 2B**) were fitted according to the proposed model (Materials and Methods) (**Fig. 8**). The obtained upper limit for the apparent dissociation constant is *K_d_app* = 397±72 µM. On the other hand, by assuming that the 13% helical content is distributed throughout the entire cytosolic domain, K*_u_* ∼6.7 and the smallest resulting value of K*_d_app* is ∼132 µM for V45 (**Fig. 8**). The upper and lower limits of *K_d_app* are in the order of magnitude of the largest dissociation constant values found for other BH3-peptide/prosurvival protein complexes (nM to >100 µM) [9], [16], [17].

These results are in contrast to recent studies by isothermal titration calorimetry on the binding capabilities of Diva to peptides comprising the BH3 domain of several Bcl-2 proteins [22]. These studies found that Diva does not show “substantial affinity” for any BH3-derived peptide, including Bak and Harakiri. However, previous work using co-immunoprecipitation indicates that Diva can interact with Bak [21]. Our ELISA and NMR data also show unambiguously that Diva interacts with Harakiri and that the interaction is specific as binding involves a particular region in the 3D structure of Diva (**Fig. 3A, D**). The observed differences could be related to the systems and conditions used in the different experiments. Particularly, the isothermal calorimetry titrations reached Harakiri BH3-peptide concentration of only 17.5 µM, whereas the upper limit for the apparent *K_d_* of our studies is ∼400 µM. Moreover, the BH3 peptide from Harakiri used in the isothermal titration calorimetry is 26 residues long. This is shorter than Hrk_medium, which already shows very weak binding relative to Hrk_ΔTM. Other reports have related the length of peptides comprising Harakiri's BH3 domain to differential binding affinity for Bcl-2 and Bcl-X_L_, with longer peptides (26 vs. 20 amino acids) showing stronger interactions [**17**].

The isothermal titration calorimetry results are also in contrast with the structural similarity between Diva and other Bcl-2 proteins [22]. Mutations in Diva relative to other members of the Bcl-2 family in two conserved residues (G88S, R89Q) of the BH1 domain that forms part of the hydrophobic cleft have been suggested to be responsible for the non-detected binding [22]. By contrast, our NMR data clearly show that Diva uses this hydrophobic groove, including the BH1 domain (**Fig. 2C**), to interact with Harakiri. Particularly, significant changes in the chemical shift of S88 (**Fig. 2B** **,** **Fig. 8**) and Q89 (**Fig. 2C**) upon the interaction with Hrk_ΔTM were observed, indicating that these residues are perturbed by the interaction.

### Comparison of Diva/Harakiri affinity to other heterodimers in the Bcl-2 family

Dissociation constant values of direct interactions between antiapoptotic Bcl-2 proteins and BH3-derived peptides measured in vitro by fluorescence polarization have been found to correlate with the apoptotic response [17]. Complexes with *K_d_* in the nanomolar range clearly show stronger killing activity than weaker interactions [17]. However, heterodimers with affinity in the micromolar range (>2.5 µM) are also functional [17]. For instance, the BH3 domains of the BH3-only proteins Noxa A and Noxa B with significant affinity (*K_d_* ∼20–30 nM) only for antiapoptotic Mcl-1, show comparable killing activity in the presence of Mcl-1 or Bcl-2, albeit the measured *K_d_* for the latter is >2.5 µM [17]. Similar results were found for the BH3 domain of the BH3-only protein Bik, which shows equivalent killing activity in the presence of Mcl-1 and BFL-1 although the affinity for both antiapoptotic proteins is significantly different (*K_d_* = 109 nM for Mcl-1 and >2.5 µM for BFL-1) [17].

More relevant to the Diva/Harakiri complex are the results found for the BH3 domain of Harakiri, which shows killing activity in the presence of Bcl-2 for which the measured *K_d_* is >2.5 µM [17]. In addition, some discrepant results on the binding capabilities of Harakiri for antiapoptotic Bcl-2 members have been reported. For example, IC_50_ values ∼50 nM, measured by competition assays using surface plasmon resonance, have been found for the interaction between the BH3 domain of Harakiri and the antiapoptotic proteins Bcl-w and BFL-1 [16]. In contrast, *K_d_* values >2.5 µM in the direct binding affinity studies have been reported for the same proteins [17]. These discrepancies could result from the different techniques, conditions and systems used.

In particular, it is noteworthy that 4, out of the 9 BH3-only proteins known, show a transmembrane domain. Specifically, Harakiri [18] and Diva [21] have been found predominantly in the membrane, and thus they are expected in vivo to interact while anchored to the membrane. The differences between in vivo and in vitro binding conditions will likely be larger for those BH3-only members with a transmembrane domain, resulting in dissociation constants values measured in vitro that are significantly different in vivo. For example, higher protein local concentration at the membrane surface and restricted mobility resulting from membrane anchoring could increase binding affinity. Therefore, the K*_d_app* value reported above for the Diva/Harakiri heterodimer is only a rough estimate of the “real” dissociation constant between these proteins, which most likely decreases under in vivo conditions.

## Discussion

The ELISA and NMR data herein reported consistently demonstrate that the Bcl-2 members Diva and Harakiri are able to interact in vitro. Moreover, the NMR results indicate that the interaction is specific involving in Diva the same hydrophobic cleft observed in all of the reported 3D structures of other Bcl-2 complexes. No information on the interaction between Diva and Harakiri has been previously reported. Thus, further studies are necessary to test whether the Diva/Harakiri complex is functionally relevant in apoptosis. Nevertheless, from the biophysical and structural perspective our results indicate that Diva is structurally suited to function as other negative regulators of cell death, in contrast to recent binding studies suggesting that the structure of Diva reveals a functionally divergent protein [22].

In addition, we show that the full-length cytosolic domain of Harakiri is intrinsically disordered with residual α-helical structure. Therefore, we propose that Harakiri folds as an α-helix upon complex formation, as previously suggested for the interaction between the BH3-only member Bim and the antiapoptotic protein Bcl-w [10]. Our data also indicate that the cytosolic domain of Harakiri binds Diva with higher affinity than the shorter constructs. However, Diva shows the same interacting surface for both Hrk_ΔTM and Hrk_medium, suggesting that factors other than those pertinent to intermolecular interactions within the interacting area are playing a role in binding. The influence on binding of disordered regions outside the interacting interface has been theoretically predicted [32] and experimentally observed before [33]. Intrinsically disordered proteins can follow different binding mechanisms in which preformed elements of secondary structure, together with concomitant folding and flexibility in the unbound and bound state can play important roles [34]. Thus, further mechanistic studies are necessary to identify the factors responsible for the affinity differences of the Harakiri constructs. Nevertheless, our results suggest that studies on fragments longer than the typically 25-residue BH3 peptides will help to better understand Bcl-2 interactions.

## Materials and Methods

### Protein production

The c-DNA fragment encoding for residues 1–160 of mouse Diva (lacking its putative 30-residue long transmembrane α-helix) was cloned into the NcoI and HindIII restriction sites of pBAT4 expression vector [35], which was transformed in BL21(DE3) E. Coli strain (Novagen). Bacteria were grown at 37°C and protein expression was induced at OD_600_ of 0.6–0.7 for 6 h at 30°C by adding 1 mM IPTG (isopropyl-β-D-thiogalacto-pyranoside). Uniformly ^15^N- and ^13^C-labeled Diva was produced using ^13^C_6_-D-glucose and ^15^NH_4_Cl (Spectra Stable Isotopes) as sole carbon and nitrogen sources, respectively. The cells were harvested by centrifugation and resuspended in a buffer containing 50 mM sodium acetate at pH 5.4, 50 mM NaCl, 0.1 mM protease inhibitor cocktail (Sigma) and 1 mM TCEP (Tris(2-carboxyethyl)phosphine). Cells were lysed by sonication at 4°C and centrifuged at 25000 rpm for 30 minutes. The soluble protein was purified by cation exchange chromatography using an SP sepharose Fast Flow column (GE Healthcare). A second purification step was necessary using reverse phase chromatography in water-acetonitrile mixtures, followed by lyophilization of the protein solution.

### Peptide synthesis

The full-length cytosolic domain of human Harakiri, residues 1_59 (Hrk_ΔTM), and fragments 22–53 (Hrk_medium) and 33–47 (Hrk_BH3) were synthesized and purified by CASLO Laboratory (Denmark). The purity (>95%) and molecular weight were confirmed by liquid chromatography and mass spectrometry, respectively. Hrk_medium and Hrk_BH3 are protected by a C-terminal amide.

### Enzyme-linked immunosorbent assays (ELISA)

Microplates (Costar Ltd., US) were coated with 50 µl of Diva in PBS at a concentration of 7.5 µg/ml. After overnight incubation at 4°C plates were washed three times with distilled water and then blocked with 2.5% BSA (Sigma-Aldrich, Spain) in PBS for 2 h at 37°C. Solutions of Harakiri fragments were prepared at fixed initial peptide concentrations (derived from absorbance measurements) containing PBS, 2.5% BSA and 0.1% Tween to avoid aggregation and non-specific binding. Samples were loaded and incubated overnight at 4°C. BSA at 2.5% in PBS was used as negative control. After thorough wash (6 times) with distilled water and 0.1% Tween, bound Harakiri fragments were detected by a polyclonal rabbit anti-Harakiri BH3 domain antiserum (1∶1000) (Abcam, UK). Following a streptavidin peroxidase conjugate anti-rabbit Ig (1∶1000) (Dako) as secondary antibody, the peroxidase activity was detected by the addition of 3,3′,5,5′-tetramethylbenzidine dihydrochloride peroxidase substrate (Sigma). Colour development was then allowed to proceed for 15 min at room temperature and stopped adding 0.1 M H_2_SO_4_. The optical density was read at 450 nm on an ELISA plate reader (Labsystems Multiskan BICHROMATIC). All assays were done in duplicate. The absorbance of the control is within typical background values in ELISA experiments indicating close to optimum conditions for binding level detection.

### NMR experiments

NMR samples were prepared at 0.1–0.22 mM ^13^C,^15^N-labeled Diva in 10% ^2^H_2_O/H_2_O containing 20 mM deuterium-enriched sodium acetate buffer at pH 5.4, 5 mM ^2^H_15_-TCEP and 0.1 mM NaN_3_. NMR data were acquired at 35°C on a Bruker Avance III 600 MHz spectrometer with a triple resonance tri-axial gradient probe. Amide ^1^H-^15^N chemical shifts were obtained using [^1^H-^15^N]-HSQC experiments [25] that were processed with NMRPipe [36] and analyzed with PIPP [37]. Diva samples were titrated with stock solutions of unlabeled Hrk_ΔTM, Hrk_medium and Hrk_BH3 in the same buffer used for Diva. Protein and peptide concentrations were derived from absorbance measurements. For each titration point [^1^H-^15^N]-HSQC experiments were acquired to monitor chemical shifts changes in Diva upon Harakiri fragment addition. [^1^H-^1^H]-TOCSY experiments on Hrk_ΔTM in the presence and absence of Diva were acquired with a mixing time of 80 ms. Hrk_ΔTM is in molar excess relative to Diva in the mixture (0.85 mM/0.12 mM, Hrk_ΔTM/Diva) for the observation in the [^1^H-^1^H]-TOCSY spectrum of signals from Hrk_ΔTM alone.

### Circular dichroism experiments

Hrk_ΔTM was dissolved in 20 mM sodium phosphate buffer at pH 5.8 in the presence and absence of 35% (v/v) trifluoroethanol (TFE). The concentration of Hrk_ΔTM was 23 µM in the buffer without TFE and 44 µM in TFE. The concentration was calculated by measuring absorbance at 280 nm. CD measurements were acquired at room temperature using a JASCO model J-815 spectropolarimeter with a 1 mm cuvette. The CD signal at 222 nm was converted to mean residue ellipticity ([*θ*]_obs_) after subtracting the blank using the equation:
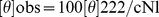
(1)where c is the peptide concentration (in mM), N is the number of peptide residues and l is the path-length (in cm).

The percentage of α-helical population was determined using the following equation:
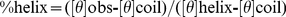
(2)where [*θ*]_helix_ is the mean residue ellipticity of a complete helix, i.e., -42,500(1-(3/N)), and [*θ*]_coil_ is the ellipticity of a random coil, i.e. +640 [38], [39].

### Apparent dissociation constant measurement

Exchange between the free and bound forms of Diva is fast on the NMR time scale and thus the observed chemical shifts (δ) are weight-averaged relative to the free and bound fractions:
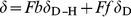
(3)


Where *Fb* is the fraction of bound Diva, *Ff* is the fraction of free Diva, δ_D_H_ is the chemical shift of Diva bound to Hrk_ΔTM and δ_D_ is the chemical shift of free Diva. Since *Fb*+*Ff* = 1, equation (3) becomes;
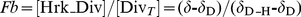
(4)where [Hrk_Div] and [Div*_T_*] are the concentration of Diva bound to Hrk_ΔTM and the total concentration of Diva, respectively. (δ_D_H_ - δ_D_) is the chemical shift change at infinite concentration of Hrk_ΔTM.

Previously reported studies on coupled folding and binding have shown that the binding process might no be adequately represented by two states [31]. Therefore, for a more realistic description of the binding of Hrk_ΔTM to Diva taking into account that Hrk_ΔTM is largely unstructured in the unbound form and likely folds upon binding, we have assumed the simplest model in which Hrk_ΔTM folding/unfolding equilibrium is related to binding as follows:
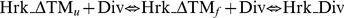
(5)where Hrk_ΔTM*_u_* and Hrk_ΔTM*_f_* are the unfolded and folded forms of Hrk_ΔTM, respectively. Div is the unbound form of Diva and Hrk_Div is the complex.

The equilibrium constants of the model shown in equation (5) are:
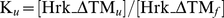
(6)

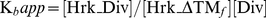
(7)


K*_u_* and K*_b_app* are the unfolding and apparent binding constants, respectively.

In addition, the total concentration of Diva and Hrk_ΔTM are: 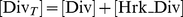
(8)

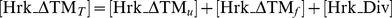
(9)where [Div*_T_*] and [Hrk_ΔTM*_T_*] are the total concentration of Diva and Hrk_ΔTM at each titration point. Substituting equation (9) in (6):
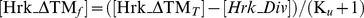
(10)


Substituting equations (4), (8) and (10) in equation (7):
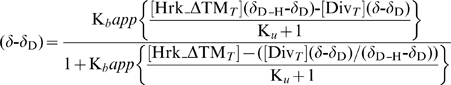
(11)


There are three unknowns in equation (11), i.e., (δ_D_H_ - δ_D_), K*_b_app* and K*_u_*. To fit the data properly we fixed the value of K*_u_*, which can be estimated from the CD experiments (**Fig. 5**). Upper and lower limits of K*_u_* depend on the number of residues assumed to adopt the helical conformation and derive in upper and lower limits for K*_b_app*. For the K*_u_* lower limit it was assumed that the helical population is localized in the critical binding region in Harakiri (residues 22–53). Thus, out of the 59 residues in Hrk_ΔTM, the 32 residues of Hrk_medium shown to be sufficient for the interaction fold into an α-helix. This assumption leads to folded and unfolded populations of 24% and 76%, respectively, and thus K*_u_* ∼3.1. By fixing this parameter the changes in chemical shift resulting from the titration of the total of 10 signals shown in **Fig. 2B** were fitted to equation (11) using the software GRACE (http://plasma-gate.weizmann.ac.il/Grace). The individual fits and calculated K*_d_app* are shown in **Fig. 8**. A global upper limit for K*_d_app* is obtained from the average of the 10 values (397±72 µM). For the K*_u_* upper limit it was assumed that all residues populate the helical conformation. Thus, the folded and unfolded populations are 13% and 87%, respectively and K*_u_* is ∼6.7. Fixing this parameter the lowest K*_d_app* value is 132 µM for V45.

### Molecular docking calculations of Diva/Harakiri complex

The program I-TASSER (Protein structure and function prediction based on iterative TASSER simulations) [40] was used to produce a 3D model of Hrk_ΔTM (59 residues). For a better representation of Hrk_ΔTM, the structure of the longest (35 residues) reported α-helical peptide comprising the BH3 region of a BH3-only protein (Bid) complexed to a prosurvival Bcl-2 protein (Mcl-1) [28] was used as template. The five predicted structures with the highest scores consistently show an α-helix spanning Harakiri residues 23 to 57. In contrast, loops of different lengths are predicted from residues 1 to 22. Molecular docking was carried out with the software HADDOCK (High Ambiguity Driven biomolecular DOCKing based on biochemical and/or biophysical information) [41] using the 3D structure of Diva [22] and the structure of Hrk_ΔTM with the highest score from I-TASSER. The online HADDOCK server was used to build a model of the Diva/Hrk_ΔTM heterodimer. HADDOCK uses residues involved in the interaction (so-called “active” residues) to create a list of ambiguous interaction restraints used in the docking calculation. These restraints set intermolecular interatom upper distance limits of 2 Å for all atoms of residues input as “active”. “Passive” residues, which do not directly participate in the interaction and surround active ones, can also be incorporated. Residues in helices 2, 3, 4, 5 and 8 with chemical shift perturbations larger than 0.05 ppm and with >20% of the total surface solvent exposed were included as “active” for the molecular docking. No “passive” residues were defined. Solvent accessibility was calculated with MOLMOL [42]. The list of “active” residues used and the corresponding percentage of solvent accessible surface is: R43 (33%), R47 (36%), Q48 (26%), Q51 (38%), H53 (24%), E55 (37%), F56 (42%), S59 (27%), S63 (25%), R64 (51%), K77 (47%), S88 (23%), D152 (37%), R156 (50%), F157 (34%), K159 (53%). For HrK_ΔTM, residues from 22–53 known to be critical for the interaction from the NMR and ELISA data were included as “active” residues. No “passive” residues were defined. HADDOCK default parameters were accepted for the docking protocol. No other restraints were incorporated by the user besides the residues specified above. The three resulting HADDOCK clusters of structures with highest scores out of 1000 calculated were inspected and compared to known antiapoptotic protein/BH3-peptide 3D complexes. The relative orientation of both molecules in the best structure of cluster 3 is closer to other known complexes and thus was selected as the representative structure.
